# Conducting Polymers and Their Applications in Diabetes Management

**DOI:** 10.3390/s16111787

**Published:** 2016-10-26

**Authors:** Yu Zhao, Luyao Cao, Lanlan Li, Wen Cheng, Liangliang Xu, Xinyu Ping, Lijia Pan, Yi Shi

**Affiliations:** 1 School of Electronic Science and Engineering, Collaborative Innovation Center of Advanced Microstructures, Nanjing University, Nanjing 210093, China; chemdream@foxmail.com (Y.Z.); 131242002@smail.nju.edu.cn (L.C.); lanlan_li@foxmail.com (L.L.); chengwen_ese@outlook.com (W.C.); 2 Nanjing Foreign Language School, Nanjing 210008, China; dg1523015@smail.nju.edu.cn (L.X.); flashlight123@126.com (X.P.)

**Keywords:** conducting polymers, glucose biosensor, diabetes, medical controlled release

## Abstract

Advances in conducting polymers (CPs) have promoted the development of diabetic monitoring and treatment, which is of great significance in human healthcare and modern medicine. CPs are special polymers with physical and electrochemical features resembling metals, inorganic semiconductors and non-conducting polymers. To improve and extend their properties, the fabrication of CPs and CP composites has attracted intensive attention in recent decades. Some CPs are biocompatible and suitable for biomedical use. Thus, the intriguing properties of CPs make wearable, noninvasive, continuous diabetes managing devices and other potential applications in diabetes possible in the near future. To highlight the recent advances of CPs and their derived materials (especially in conducting polymer hydrogels), here we discuss their fabrication and characterization, review the current state-of-the-art research in diabetes management based on these materials and describe current challenges as well as future potential research directions.

## 1. Introduction

Diabetes mellitus, commonly referred to as diabetes, is a big threat to human health globally. It is a disease characterized by high blood glucose levels (BGLs), which can cause severe damage to the eyes, kidneys, and nerves. In addition, it can also cause heart disease, strokes and even the need to remove limbs. Such complications can however be alleviated if controlled duly and effectively. Metabolites such as glucose are important indicators in diabetes management. The existing standard therapy for patients with insulin-dependent diabetes (type 1) and advanced patients with non-insulin-dependent diabetes (type 2) diabetics includes everyday insulin injections, and frequent fingerstick calibrations to monitor BGLs [1,2]. Frequent injections and fingerstick calibrations are painful and has a high risk of infection as a result of exposure to the environment and the low immunity of diabetics, hence a more accurate, riskless and less painful method is highly desirable. Therefore, intense scholarly interest has been aroused concerning the detection, tracking, and control of metabolites using optical, electromagnetic or electrochemical sensors. 

**Figure 1:**
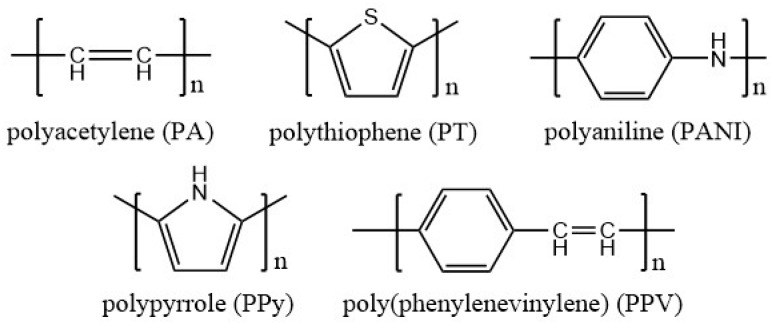
Molecular structures of typical conductive polymers.

Conducting polymers (CPs), the so-called “fourth generation of polymeric materials” [3], can provide effective methods for the diagnosis and treatment of diabetes. Many scientists have been working on these exciting newly-emerging materials, obtaining an extensive range of materials from insulators to metals. CPs show nearly no conductivity in the neutral state. In 1976, the conductivity in polymers such as polyacetylene doped p-type dopants (oxidants) was first discovered by MacDiarmid, Shirakawa, and Heeger [4,5], to be followed by the discovery of n-type dopants (reducing agents) in 1978 [6]. Heeger, MacDiarmid and Shirakawa were awarded the Nobel Prize in Chemistry in 2000 “for the discovery and development of conductive polymers”. A new class of conducting polymers, intrinsically conducting polymers (ICPs), containing monomers capable of acquiring one or more positive or negative charges through oxidation or reduction, which leads to conductivity, was found later. Two other widely-used classes of conducting polymers are redox polymers and ionically conducting polymers (polymer/salt electrolytes). Compared to ICPs, redox polymers are less conductive. As for ionically conducting polymers, their conductivity is due to the flow of ions. Their applications in sensors are limited by the low ionic conductivity at room temperature and time-dependent increase in resistance of the polymer electrolyte. Nowadays, more conducting polymers with desirable physical properties are being created, which augurs a promising future in diabetes monitoring and treatment. Some popular and useful conductive polymers are polyacetylene (PA), polythiophene (PT), polyaniline (PANI), polypyrrole (PPy), poly(phenylenevinylene) (PPV), seen as Figure 1.

The application of nanostructured CPs to diabetes management holds a number of potential advantages, such as targeting small particular areas of cells and analysis of small amount of analytes [7]. 

Conducting polymers display remarkable properties resembling metals and inorganic semiconductors, as well as polymers. The ability to transfer electrons produced by bio-chemical processes enable these polymers to be extensively applied in biosensors. Via redox reaction or doping processes, the physical and chemical properties of conducting polymers are tunable, which can cater to diverse demands. Conducting polymers have excellent biocompatibility [8]. They can provide advantageous interfaces for bio-electrodes owing to their hybrid conducting mechanics, combining both electron and ionic charge carriers. Besides CPs, hydrogels are a highly cross-linked network of polymer chains with three-dimensional (3D) hierarchical structures. Some composites combining conducting polymers and hydrogels are known as conducting polymer hydrogels (CPHs). Owing to their porous microstructures, CPHs have high surface area and can absorb great quantities of water with high tensile strength and low microbial penetration [2]. 

**Figure 2:**
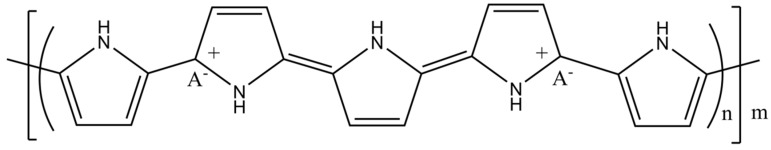
Doping of conducting polymer (polypyrrole, PPy) with ionic species A.

Conducting polymers such as polypyrrole (PPy) have an inherent instability, as a result of alternative formation of single and double bonds when the monomers polymerize. Pi-bonded electronic delocalization across the conjugated backbone, provides a “highway” for mobile charge carriers introduced through doping [9,10]. Hence, the highly tunable physical and electrochemical properties are determined by the structure of the polymer networks and the species and the concentration of the dopant. Figure 2 shows a typical alternate PPy structure of single and double bonds with dopant ion A.

**Figure 3:**
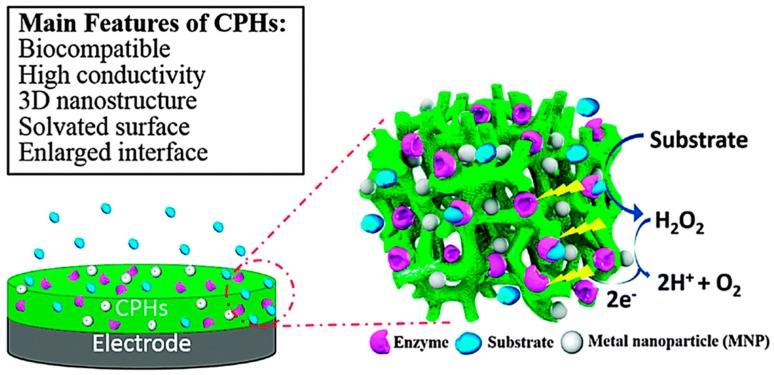
Schematic of a typical example of an electrochemical biosensor based on CPHs and a list of the main properties of CPHs. Reprinted with permission from [16]. Copyright (2015) The Royal Society of Chemistry.

The conventional syntheses of conducting polymers can be divided into two methods, electrodeposition and chemical synthesis. Electrodeposition is conventionally used for coating microelectrodes, such as metallic electrodes and carbon materials [11,12,13]. However, this method provides poor control over the structure of the resulting polymers. Random-chained polymers are hardly soluble, not easy to process melt and post-process. Compared to electrodeposition, chemical synthesis enables mass production with enhanced processability at the expense of efficiency and simplicity. Moreover, the polymers resulting from chemical synthesis have low electrical conductivity, and require post-processes to achieve the target conductivity. Some new syntheses involving self-assembly, deposition in a hydrogel matrix template [10], or multivalent metallic ion crosslinking, have been applied to the fabrication of CPHs. In addition, our group introduced a general CPH synthesis process by crosslinking CPs with multi-valent acids [14,15]. Figure 3 shows the typical structure of CPHs and main features combining both CPs and hydrogels.

Conducting polymers and hydrogels are “intelligent” materials as they can endure abrupt external physical or chemical changes. Hence, they have promoted the development of novel glucose measurement and insulin delivery modalities which hold the potential to dramatically improve the quality of life for diabetics. DiSanto et al. [7] have reviewed advances in nanotechnology for diabetes treatment. Li et al. [16] reviewed rational design and applications of conducting polymer hydrogels as electrochemical biosensors. Advances of glucose monitoring will benefit more accurate drug dosing and diabetes control systems [7]. Improvements of nanomedicine can encourage novel sensors with advanced continuity, noninvasiveness [17] and usability [18,19,20], as well as more continuous and automatic “closed-loop” insulin release systems [18,21].

## 2. Applications of Conducting Polymers in the Monitoring of Diabetes

In 1962, Clark and Lyons [22] developed the first biosensor by integrating an enzyme, glucose oxidase (GOx), into an electrode. Since then, tremendous progress has been made in the development of biosensors for use in the detection and quantization of metabolites. Numerous glucose biosensors have been invented, such as optical [23], electromagnetic [24] or electrochemical sensors [25].

### 2.1. Conducting-Polymer-Based Glucose Sensors

Conducting polymers are highly conjugated pi-bonded polymers, with special conductivity as a result of the delocalization of electrons [9]. In addition to the conventional polymer properties such as flexibility and processability, CPs possess some unique optical and electrical properties like metals and inorganic semiconductors [26], which allow them to become robust platforms of biosensors or bioelectrodes for electron transfer and immobilization of biomolecules or inorganic nanoparticles [27], such as glucose oxidase (GOx) [28], metal nanoparticles [29,30], and carbon nanotubes [31,32]. To fabricate the immobilization matrices, many techniques have been applied, such as crosslinking, entrapment, physical adsorption, and covalent bonding [33,34,35,36,37]. Furthermore, CP composites, such as CP composites of chitosan, glucose oxidase, metal nanoparticles, metal salts, metal oxide particles, and carbon nanotubes, are wildly used in the fabrication of sensors. Both immobilization and incorporation of second component can enhance the functionality by catalyzing a reaction, increasing the conductivity, or enhancing biocompatibility [38].

Conducting polymers have been used in many glucose sensors with excellent properties. Many glucose biosensors use confined/immobilized enzymes to form a selective layer on CP structures [39]. The 1st generation biosensors measure a product, co-product or co-substrate of the enzyme reaction directly [22], however, 1st generation biosensors have the disadvantages of needing high redox potential, susceptibility to oxygen partial pressure, etc. To overcome these drawbacks, 2nd generation biosensors monitor the current of electron transfer mediator (ETM) between electrode and the redox center of enzyme. Adeloju et al. [40] have studied a PPy–GOx film in a supporting electrolyte-free monomer solution, as a potentiometric glucose biosensor. Mediator free 3rd generation biosensors detect direct electrical transfers between an electrode and the redox active center. Forzani et al. [28] presented a 3rd [41] glucose biosensor using conducting polymer/enzyme nanojunctions arrays. Each nanojunction is formed by bridging a pair of nanoelectrodes separated by a small gap (20–60 nm) with PANI/GOx. Due to the extremely small size of the nanojunction and large surface area of PANI, the sensor does not need redox mediators and has a fast response. PANI exhibits two redox couples in the right potential range to facilitate a direct electrical communication between the enzyme and the electrode and thus acts as a self-contained mediator without additional diffusional mediators [42]. Consequently, it can overcome the drawbacks of 2nd generation biosensors, such as the leaching susceptibility of the soluble mediator into the sample solution, and the diffusion barrier of the mediator between enzyme/electrode interface, and provide long-term stability [41,42].

**Figure 4:**
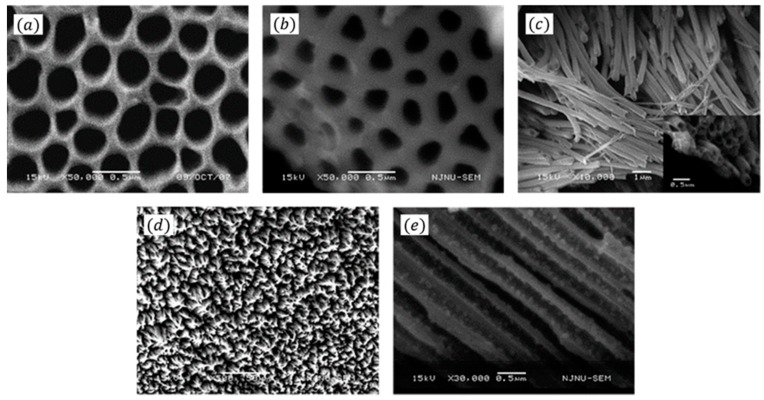
(**a**,**b**) SEM image of the AAO membrane before (a) and after (b) formation of PANI nanotubes in the pores of the template. (**c**,**d**) SEM images of polyaniline nanotubes obtained by etching away the AAO membrane. (**e**) Cross-sectional image of the polyaniline nanotube after loading GOx on the inner wall of the nanotube. Reprinted with permission from [43]. Copyright (2009) American Chemical Society.

Wang et al. [43] developed a glucose biosensor by electrochemically entrapping GOx onto the inner wall of a polyaniline nanotubes matrix (nanoPANi) with a template of an anodic aluminum oxide (AAO) membrane, seen in Figure 4. It has been indicated that a direct electron transfer reaction has taken place, and the cyclic voltammogram shows a pair of well-defined and nearly symmetric redox peaks. The electron can be directly transferred by GOx without additional mediators or cofactors. Moreover, the sensor have great anti-interference performance against common interfering species in blood, such as ascorbic acid, uric acid, and 4-acetamidophenol, due to the low detection potential (−0.3 V vs. SCE). 

The sensitivity and detection limit of CP biosensors depend on the background current presented by the polymer [3]. Incorporation of metal particles can efficiently improve the conductivity [44,45]. Xian et al. [44] reported a glucose biosensor using a composite of Au nanoparticles (AuNPs)/polyaniline (PANI) nanofibers with immobilization of GOx and Nafion on the surface of nanocomposite. The biosensor had good sensitivity and selectivity for glucose and good operational stability (over 2 weeks). Mazeiko et al. [46] have shown that AuNPs can facilitate electron transfer and they can amplify amperometric signals on electrodes. Zhou et al. [45] investigated the application of platinum nanoparticles (PtNPs) on glucose biosensors with GOx/PMPD/Pt/PANI electrodes.

Carbon nanotubes (CNTs), discovered in 1991 [47], have become popular owing to their unique structure, high surface-to-volume ratio and high chemical stability. Conducting polymers are capable of acting as matrices for the dispersion of metallic particles or biomolecules on the surface of CNTs [48,49,50,51]. Xu et al. [52] prepared an amperometric glucose biosensor based on composites of multi-wall carbon nanotubes coated with PANI, dendrimer-encapsulated Pt nanoparticles (Pt-DENs), and self-assembling GOx.

Graphene is a two-dimensional sheet providing fast electron transfer and excellent electrocatalytic characteristics. Graphene has prospective application in synthesis with CPs to construct biosensors [53]. Xu et al. [54] developed a GOx/graphene/PANI/gold nanoparticles (AuNPs) with GOx adsorbed in graphene/PANI/AuNPs nanocomposite-modified glassy carbon electrode (GCE). The adsorbed GOD displayed a pair of well-defined quasi-reversible redox peaks with a formal potential of −0.477 V (vs. SCE) and an apparent electron transfer rate constant (*k_s_*) of 4.8 s^−1^ in 0.1 M pH 7.0 PBS solution.

Miniaturization of glucose sensors is a new requirement. Mini sensors are portable and wearable with low consumption of sample. The micro-electro-mechanical system (MEMS) technique can facilitate the fabrication of mini sensors. Xu et al. [55] designed a glucose sensor based on high aspect ratio carbon post-microarrays. The carbon post-microarrays were fabricated by carbon-microelectromechanical systems (C-MEMS) technology. Enzymes are immobilized onto the carbon post-electrodes by co-deposition of GOx and electrochemically polymerized PPy.

As mentioned, CPs offer many extraordinary features suitable for diabetes measurement. It is acknowledged that the two common properties desired for all biomedical applications are biocompatibility and redox stability, and thereby for biosensors, hydrophobicity, reactive functionalities, as well as conductivity really matter [56]. However some CPs are hydrophobic so that they can be damaged and diffuse the entrapped proteins [56]. More hydrophilic polymers have been proposed in biosensors

### 2.2. Conducting-Polymer-Hydrogel-Based Glucose Sensors

Conducting polymer hydrogels, composite polymer matrices of conducting polymers and hydrogels, have high water content, providing a more biocompatible environment for in vivo glucose monitoring and immobilization of biomolecules [8]. Conductive networks of CPs provide pathways for the transfer of electrons in CPHs [57]. Porous 3D CPH nanostructures possess large surface areas and decrease the diffusion of biomolecules [58]. Heller introduced biosensors based on redox hydrogels and ionically cross-linked CPHs, and proposed some of their actual applications, particularly in BGL-monitoring electrodes for diabetics [58,59]. 

**Figure 5:**
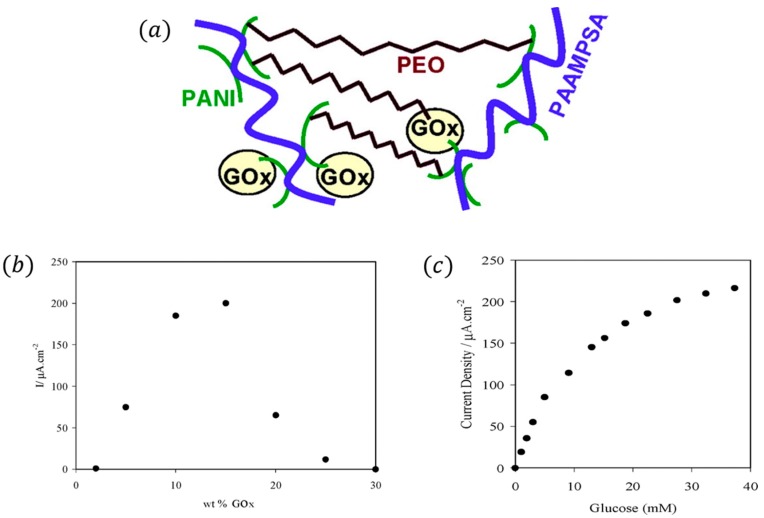
(**a**) A conducting cross-linked PANI-based redox hydrogel. (**b**) Dependence of the current density on the glucose oxidase weight percentage, with glucose concentration maintained at 32 mM. (**c**) Dependence of the steady-state current density on the glucose concentration for an electrode set at +0.3 V vs. Ag/AgCl. Reprinted with permission from [60]. Copyright (2007) American Chemical Society.

Heller et al. [60] formed a PANI-based glucose-permeable CPH in one step at pH 7.2. Cross-linking of acid-templated PANI with a water-soluble diepoxide, poly(ethylene glycol diglycidyl ether), and immobilization of GOx in the hydrogel, occurs in one step, seen as Figure 5a. The current density of electrocatalytic oxidation of glucose is at a steady rate, 225 μA·cm^−2^ at 0.3 V vs. Ag/AgCl, as seen in Figure 5b,c. Åsberg et al. [61] used poly(3,4-ethylenedioxythiophene)/poly(styrene sulfonate) aqueous dispersion (PEDOT/PSS) to build a CPH matrix. After immobilization of appropriate biomolecules this constitutes a hydrogel bioelectrode. The open hydrogel structure makes analytes penetrate easier into the matrix electrode.

Liu et al. [62] investigated an organic thin film transistor (OTFT)-based glucose sensor. GOx was immobilized in a PEDOT–PSS conducting polymer film by a simple spin-coating method and was entrapped in the polymer matrix during electrochemical polymerization. Yang et al. [63] fabricated a biosensor based on an inverse opal PANI doped with poly(styrene sulfonate) by electrochemistry. PANI/PSS is prepared using silica spheres as sacrificial templates. The inverse opal PANI 3D structure offers a platform for increasing the amount of GOx contacting the electrode, hence, facilitating direct electron transfer between the enzyme and electrode, resulting in higher sensitivity and short response times.

Nano-porous templates can improve the enzyme loading efficiency, which provides an increased surface area for absorption. Enhanced adsorption will reduce the amount of wasted enzyme, and thus the production cost will be reduced. Ekanayake et al. [64] discussed the feasibility of enzyme entrapment by physical adsorption and described a novel amperometric biosensor with increased glucose adsorption. The sensor is based on a polypyrrole (PPy) nanotube array deposited on a Pt plated nano-porous alumina substrate. Nano-porous alumina discs were used to fabricate the electrodes. A PPy/PF6-film comprising of nanotube array was synthesized. The immobilization was done by physical adsorption of GOx on each electrode. The synthesized nanotube arrays were characterized by a galvanostatic electrochemical technique. The calculated value of the apparent Michaelis–Menten constant (Km) was 7.01 mM.

Li et al. [65] used a new type of in situ electropolymerization method. The biological film was prepared by in situ electropolymerization of aniline onto a porous polyacrylonitrile (PAN)-coated platinum electrode in the presence of glucose oxidase. The biosensor exhibited good selectivity, sensitivity, and its BGL determinations agreed well with standard hospital analyses. The stability of the sensor is remarkable, because it showed no apparent loss of activity after 100 consecutive measurements and intermittent usage for 100 days with storage in a phosphate buffer at 4 °C.

**Figure 6:**
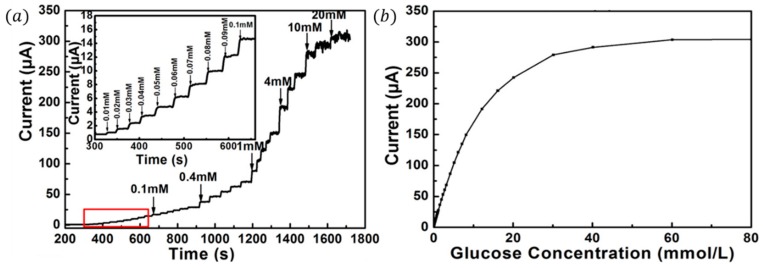
(**a**) Amperometric response of the PtNP/PANI hydrogel electrode after successive addition of glucose in 0.1 M PBS (pH = 5.6) at an applied potential of 0.56 V (the magnified part of the curve is marked with a red square); (**b**) the calibration curve for glucose concentrations from 1 μM to 80 mM. Reprinted with permission from [66]. Copyright (2013) American Chemical Society.

Reversibility, an important characteristic of desirable continuous glucose sensors, is the ability to detect both increasing and decreasing glucose concentrations. Zhai et al. [66] presented a highly sensitive and reversible glucose enzyme sensor based on Pt nanoparticles (PtNPs)-PANI hydrogel heterostructures seen as Figure 6. GOx and high-density PtNPs were immobilized into the 3D nanostructured matrix of the PANI hydrogel, where the PtNPs mediate catalysis of hydrogen peroxide and the PANI hydrogel acts as a signal conductor.

Versatile monitoring is new trend in biosensors. Li et al. [67] presented a scalable, low-cost, and versatile biosensor platform for the sensitive and rapid detection of human metabolites like uric acid, cholesterol, and triglycerides based on hierarchically nanostructured CPHs. The performance of some biosensors mentioned above are listed in Table 1.

## 3. Medical Controlled Release in Diabetes

Insulin delivery is crucial for diabetics to maintain BGLs, improve their quality of life and extend lifespans. The contemporary therapy involves the use of fingersticks several times a day to monitor BGLS or administer medicine, which causes pain, trauma, and risk of infections. Additionally, doses are based on averages volumes. Drug-delivering feedback loops, comprising macrosensors and drug delivery systems, can individualize treatment, and provide safer and more effective therapy [68]. Smart glucose-responsive “closed-loop” insulin delivery systems, an alternative to traditional insulin injections, mimic the function of pancreatic cells. Smart therapies can improve diabetes control and reduce the potential for ultralow BGLs, which is a potentially deadly consequence of excessive insulin dosing [69].

**Figure 7:**
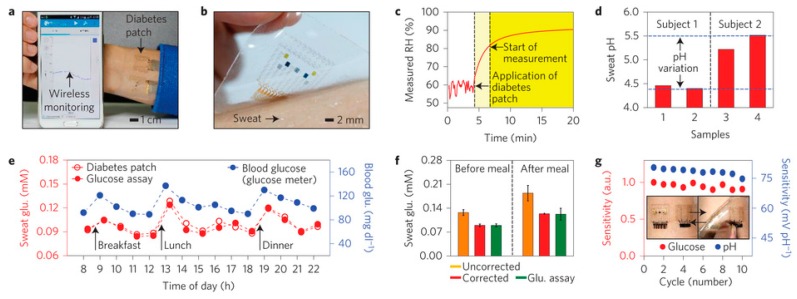

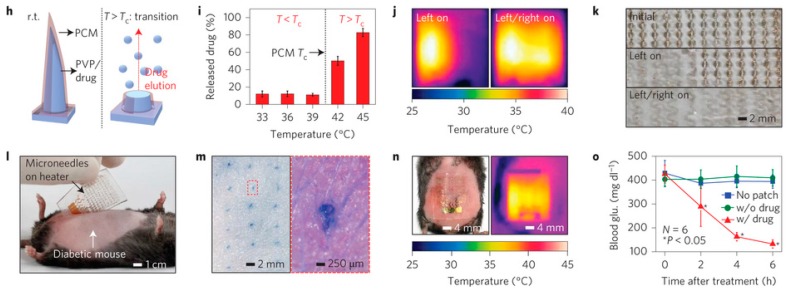
(**a**) The integrated wearable diabetes controlling and detecting system connected to a portable electrochemical analyser; (**b**) GP-hybrid electrochemical device array on the human skin with perspiration; (**c**) RH measurement by the diabetes patch. (**d**) Measurement of the pH variation in two human sweat samples from two subjects; (**e**) One-day monitoring of glucose concentrations in the sweat and blood of a human (subject 2 in d); (**f**) Comparison of the average glucose concentrations with the commercial glucose assay data in e before and after correction using the measured pH (error bars show the standard deviation); (**g**) Plots showing the stable sensitivity of the glucose and pH sensors after multiple reuses of the patch; (**h**) Schematic illustrations of bioresorbable microneedles. (**i**) Drug release from the microneedles at different temperatures (N = 3, error bars show the standard deviation); (**j**) Infrared camera images of multichannel heaters showing the stepwise drug release; (**k**) Optical images of the stepwise dissolution of the microneedles; (**l**) Image of the heater integrated with the microneedles, which is laminated on the skin near the abdomen of the db/db mouse. The hair on the skin was shaved off before treatment with the microneedles; (**m**) Optical image (left) and its magnified view (right) of the db/db mouse skin stained with trypan blue to visualize the micro-sized holes made by the penetration of the microneedles; (**n**) Optical (left) and infrared (right) camera images of the patch with the thermal actuation; (**o**) Blood glucose concentrations of db/db mice for the treated group (with the drug) and control groups (without the patch and without the drug). The error bars show the standard deviation in each group and small P values show that the results are statistically reliable. The asterisks indicate significant difference (P < 0.05) between the treated (red) and the non-treated group (blue and green) on each time point. Reprinted with permission from [70]. Copyright (2016) Macmillan Publishers Limited. All rights reserved.

Continuous and point-of-care therapy will offer convenience to patients and improve health in diabetics. Some attempts have been made, such as microneedles [70,71]. A non-invasive [17,72,73], continuous [74], long-term [74], or closed-loop [21]-based smart diabetes therapeutic system is needed for individual healthcare [1]. High conductivity, good biocompatibility, stability of chemical/physical properties, and the ability to entrap and controllably release biomolecules, enable CPs to be applied in “closed-loop” delivery devices [56,75]. Ling et al. [76] have studied dissolving polymer microneedle patches, composed of starch and gelatin, for transdermal delivery of insulin to diabetic rats. It takes 5 min after insertion into the skin for the microneedles to dissolve completely, and release the encapsulated payload into the skin. Lee et al. [70] showed a system of graphene-based sweat diabetes monitoring and feedback therapy and reduced blood glucose levels in diabetic mice, as seen in Figure 7.

**Figure 8:**
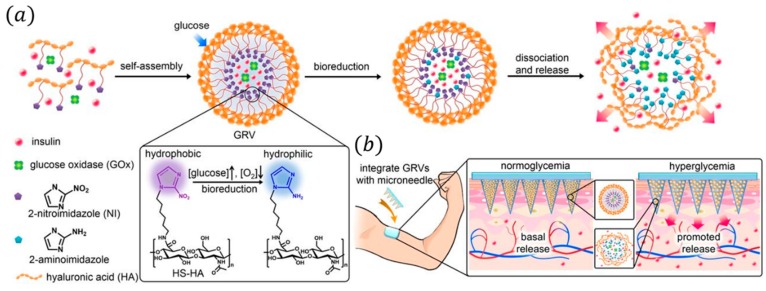
Schematic of the glucose-responsive insulin delivery system using MN-array patches. (**a**) Formation of GRVs composed of HS-HA; (**b**) Schematic of the GRV-containing MN-array insulin patch for in vivo insulin delivery triggered by a hyperglycemic state to release more insulin. Reprinted with permission from [71]. Copyright (2015) National Academy of Sciences.

Yu et al. [71] reported a “closed-loop” insulin delivery system with a painless microneedle-array patch, containing glucose-responsive vesicles (GRVs) loaded with insulin and glucose oxidase (GOx) enzyme. The GRVs are self-assembled from hypoxia-sensitive hyaluronic acid (HS-HA) conjugated with 2-nitroimidazole (NI), a hydrophobic component. The smart insulin patch regulated BGL in a mouse model of chemically induced type 1 diabetes effectively, as seen in Figure 8.

## 4. Conclusions

Conducting polymers have significant uses in many aspects of diabetic medical care, such as BGL measurement and drug release systems. Sensors can utilize CPs to become faster, more sensitive and accurate as CPs have good conductivity, and the properties are tunable by doping. CP glucose sensors have quick responses, good sensitivity, broad linear range, and low detection limits. Besides, miniature CP biosensors can meet the demands of precise detection with low sample volume and simultaneous versatile metabolic monitoring. CP biosensor devices has been designed to combine micropatterning, microfluidics, and measuring techniques. Moreover, wireless technology can be applied in CP glucose biosensors to realize wireless detection with linkage of patients/terminals and data monitor/storage/calculators. Interdisciplinary cooperation between chemistry, physics, biology, nanotechnology, material science, and electric engineering will benefit diabetes management tremendously. 

Because of their biocompatible organic components, CPs are widely used in blood glucose monitoring or drug release as coatings or conducting material for bioelectrodes. The continuous in vivo glucose meter can reduce the frequency of painful, damaging, and risky finger pricks. Additionally, continuous measurement will “close the loop” between measuring and dosing to improve the accuracy and timeliness of treatment as well as reduce healthcare costs. With good flexibility as polymers, CPs are suitable candidates for the fabrication of popular in vitro wearable devices, using sweat, tears, etc. for detection. The porous 3D nanostructures of CPs lead to large surface areas and affinity for biomolecules, which are necessary for an immobilization platform. Hence, CPs have premising and aggressive clinical applications in near future.

## Figures and Tables

**Table 1:** Comparison of the CP glucose biosensors.

Fabrication Strategy	Sensitivity (μA·mM^−1^·cm^−2^)	Response Time (s)	Detection Limit (μM)	Linear Range (mM)	Reference
GOx/PANI nanojunction	0.1	<0.2	μM-scale		[28]
GOx/nanoPANI/Pt	97.18 ± 4.62	∼3	0.3 ± 0.1	0.01–5.5	[43]
AuNPs/PANI/GOx		<5	0.5	0.001–0.8	[29]
PtNPs/PANI/GOx	96.1	3	0.7	0.01–8	[66]
GOx/PANI/PAN/Pt	67.1	<30	2	0.002–12	[65]
GOx/PPy/Al_2_O_3_/Pt	7.4	<4	30	0.5–10.4	[64]
GOx/Pt-DENs/PAni/CNT/Pt	42	5	0.5	0.001–12	[52]
GOx/Graphene/PANi/AuNPs		<8	0.6	0.004–1.12	[54]
GOx/ PEDOT/PSS	1.65	10–20		1.1–16.5	[62]
GOx/PMPD/Pt/PANI		<7		0.002–12	[45]
GOx/PANI/PDDA/Pt	64.4	<5		0.001–0.1	[63]
